# Estrogen Receptor Complex to Trigger or Delay Estrogen-Induced Apoptosis in Long-Term Estrogen Deprived Breast Cancer

**DOI:** 10.3389/fendo.2022.869562

**Published:** 2022-03-10

**Authors:** Philipp Y. Maximov, Ping Fan, Balkees Abderrahman, Ramona Curpan, V. Craig Jordan

**Affiliations:** ^1^Department of Breast Medical Oncology, University of Texas MD Anderson Cancer Center, Houston, TX, United States; ^2^Institute of Chemistry, Romanian Academy, Timisoara, Romania

**Keywords:** breast cancer, apoptosis, estrogen, estrogen receptor, structure-activity relationship

## Abstract

Antiestrogen therapy of breast cancer has been a “gold standard” of treatment of estrogen receptor (ER)-positive breast cancer for decades. Resistance to antiestrogen therapy may develop, however, a vulnerability in long-term estrogen deprived (LTED) breast cancer cells was discovered. LTED breast cancer cells may undergo estrogen-induced apoptosis within a week of treatment with estrogen *in vitro*. This phenomenon has been also validated *in vivo* and in the clinic. The molecular ER-mediated mechanism of action of estrogen-induced apoptosis was deciphered, however, the relationship between the structure of estrogenic ligands and the activity of the ER in LTED breast cancer cells remained a mystery until recently. In this review we provide an overview of the structure-activity relationship of various estrogens with different chemical structures and the modulation of estrogen-induced apoptosis in LTED breast cancer cells resistant to antihormone therapy. We provide analysis of evidence gathered over more than a decade of structure-activity relationship studies by our group on the role of the change in the conformation of the estrogen receptor and the biological activities of different classes of estrogens and the receptor as well in LTED breast cancer.

## Introduction

Breast cancer has the highest incidence in women with 252,710 new cases and 40,610 deaths in 2017 (1). The rise of breast cancer incidence over the past years is attributed to the increase in hormone receptor/estrogen receptor (ER)-positive breast cancers with a simultaneous reduction in ER-negative breast cancers (1). It is also predicted that the incidence of ER-positive breast cancers will continue to increase up to 50% from 2012 levels by 2050 (2). The activation of the ER is the driver of breast cancer progression and antihormone therapies. In the second half of the 20^th^ century i.e.: tamoxifen and aromatase inhibitors, have had a profound effect on the incidence and mortality from breast cancer worldwide (3, 4).

The first successful hormone therapy of advanced breast cancer was bilateral oophorectomy, which was based on the observation of the relationship of oophorectomy and lactation in farm animals, and became the first standard of care (5). Subsequently, the relationship between estrogen and breast cancer was proven and antiestrogenic therapy of breast cancer by synthetic antiestrogens was predicted in 1936 by Antoine Lacassagne (6, 7). However, based on the laboratory results that some carcinogenic hydrocarbons may retard the growth of tumors, Sir Alexander Haddow used high doses of estrogens (synthetic diethylstilbestrol (DES) and triphenylethylene (TPE) derivatives), hormones normally supporting the growth of breast cancer, to achieve a 30% response rate in postmenopausal patients with advanced breast cancer (8). High dose estrogen therapy became the standard of care for postmenopausal patients until tamoxifen and aromatase inhibitors were approved. Antiestrogen therapy with tamoxifen became the standard of care of ER-positive breast cancer for decades and up to present time. The reason why therapy with tamoxifen became the standard was lower systemic side effects (9, 10). This allowed the drug to transition into long-term adjuvant treatment of node-positive breast cancer patients increasing the relapse-free survival with increased duration of therapy (11). However, it was discovered that tamoxifen can induce endometrial cancer in postmenopausal women during long-term adjuvant therapy due to the presence of estrogen-like properties in the endometrium. This has been proven initially in the laboratory (12, 13) and the clinic (14, 15). Ultimately, it was shown that the mortality from endometrial cancer during tamoxifen adjuvant therapy was significantly higher in the postmenopausal group when compared to younger women (16). The results of superior control of recurrences of breast cancer in postmenopausal patients with aromatase inhibitors compared to tamoxifen (17–20) paved the way for aromatase inhibitors to become the agents of choice for the adjuvant therapy in postmenopausal patients, although with adverse effects on bone density. Tamoxifen continues to be the therapy of choice in premenopausal women.

However, resistance inevitably occurs after long-term antihormone therapy in some women. Different mechanisms of antihormone resistance have been identified, some of which will be described briefly. The main mechanisms that have been discovered are: activating mutations of the ESR1 gene encoding the ER protein (21); changes in the signaling pathways making breast cancer cell growth dependent on antiestrogenic ER ligand (tamoxifen) (22); the permanent loss of the ER (23); changes in signaling pathways enabling antihormone-resistant breast cancer cells to survive without estrogens or antiestrogens (24, 25). Interestingly, in the later variant of antihormone resistance, a vulnerability was discovered and deciphered- estrogen-induced apoptosis (26). This phenomenon of estrogen-induced apoptosis was proven in the clinic in patients after exhaustive antiestrogen therapy and acquired resistance to aromatase inhibitors (27, 28) and also explained the controversial results of the Women’s Health Initiative (WHI) (29, 30), which was designed to evaluate the benefits of the hormone replacement therapy (HRT) in postmenopausal women. The results of the WHI trial showed that the cohort of postmenopausal women with an average age over 60 with hysterectomies taking conjugated equine estrogens alone had decreased incidence and mortality from breast cancer compared to the cohort of women with intact uteri taking conjugated equine estrogens plus medroxyprogesterone acetate to prevent endometrial neoplasia. This paradoxical result can be explained by the phenomenon of estrogen-induced apoptosis (31). The aforementioned Sir Alexander Haddow, who in the 1940’s has conducted clinical trials with high-dose estrogens to treat breast cancer and in his David A. Karnofsky Award lecture has stated that in his trials the high-dose estrogen therapy was most efficient in women five years after the menopause, but the mechanisms were elusive at that time (32). It is now apparent that it takes at least 5 years of antiestrogen therapy (at least with aromatase inhibitor therapy) for breast cancer cells to develop such vulnerability to estrogens (33). In this review we will describe the mechanisms of estrogen-induced apoptosis and its dependence on the structure-function relationship of the estrogenic ligand:ER complex.

## Structure and Function of the ER

Although antiestrogenic therapy of breast cancer was predicted in the early 20^th^ century, the ER target was not identified and isolated until 1966 by Gorski and Taft (34). However, two isoforms of the receptor were subsequently identified and were labeled as ERα and ERβ, respectively (35). Both isoforms of the ER are members of the nuclear hormone receptor superfamily and bind estrogens with high affinity to modulate the activity of estrogen-dependent genes. However, only ERα is considered to be the target for treating and preventing breast cancer (36), and, thus, all further reference will be pertaining to ERα will be labelled as ER for simplicity.

The ER gene (ESR1) is located on chromosome 6q.25.1 and encodes a 595 amino acid, 66 kDa protein. The protein consists of six functional domains (37): the amino-terminal A/B domain, which contains a ligand-independent activating function-1 (AF-1) region; domain C, which contains DNA-binding zinc fingers region responsible for binding to the estrogen-dependent gene’s estrogen-response elements (ERE) in the promoter; the D domain is responsible for the nuclear localization of the protein; E domain is the ligand-binding domain (LBD), which also contains the second activating-function region (AF-2). The ligand-dependent AF-2 itself is composed of LXXLL-like motifs, which, in turn, are responsible for binding the co-activators (38). The LBD itself consists of 12α helices with H3-H12 forming the ligand-binding cavity, with H12 acting as a “lid” over the cavity. The last domain is the carboxy-terminal domain F. The inactive ER monomers are localized in the nucleus of the cells and are bound with the heat-shock proteins (HSPs) acting as chaperones. Once the ligand reaches the nucleus and binds to the ER *via* the LBD the ER : HSP complex disassociates and the receptor changes the conformation by achieving either an agonist conformation with H12 closing over the ligand-binding cavity [for example 17β-estradiol (E2)], or an antagonist conformation where H12 cannot close over the cavity, preventing the recruitment of co-activators (for example 4OHT). Once activation of the ER occurs, the receptor complexes dimerize and bind to the DNA *via* the EREs, which are composed of 15-base pair palindromic sequences. Additionally to direct DNA bringing of the receptor, the ER can also interact with other transcriptional factors, for example Fos/Jun (AP-1 responsive elements) (39), and can modulate the transcription of genes whose promoters do not contain ERE sequences. The ER can also interact with Nuclear Factor-κB (NF-κB) to inhibit transcription (40). After the activated ER-coactivator complex bound to the necessary DNA region, coactivators promote the recruitment of general transcription factors and the recruitment of DNA-polymerase II for gene transcription.

## Molecular Mechanisms of Estrogen-Induced Apoptosis

As mentioned above, estrogen-induced apoptosis was an unanticipated discovery (41) in endocrine-resistant breast cancer *in vivo* (42, 43) and *in vitro* (44, 45). Antiestrogens, tamoxifen and ICI 182,780 completely block E2-induced apoptosis (44, 45), suggesting that the ER mediates E2-induced apoptosis in these endocrine-resistant breast cancer cells (44, 45). As described above, there are two ERs: ERα and ERβ that control E2-regulated processes in women. Different actions of ERα and ERβ in E2-induced apoptosis have been differentiated using small interfering RNAs specific for respective receptors (46). Only knockdown of ERα prevents E2-induced apoptosis, confirming that E2 induces apoptosis *via* the ERα (46). To define the function of the ER-regulated nongenomic pathways precisely in E2-induced apoptosis we used a synthetic macrocompound called the estrogen-dendrimer conjugate (EDC) to specifically activate the extranuclear ER (47). As expected, EDC rapidly activates the nongenomic pathways of the ER but does not induce apoptosis in the LTED breast cancer cells, but instead EDC increases cellular proliferation. These findings demonstrate that E2 induces apoptosis only through the nuclear ER.

**Figure 1:**
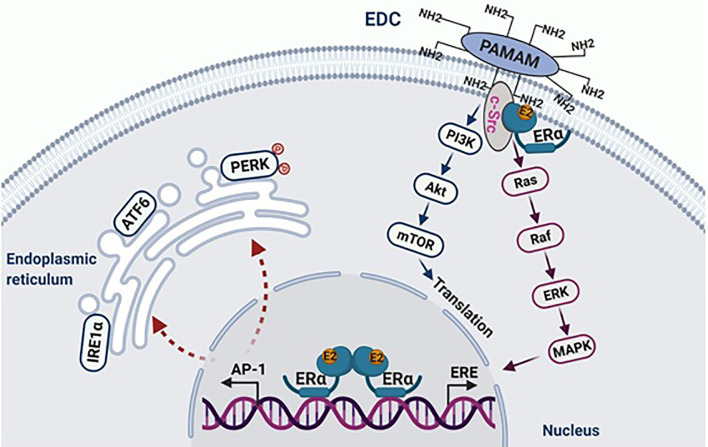
E2 initiates apoptosis through over activation of nuclear ERα. The macromolecule EDC specifically activates non-genomic pathways of ERα mediated by c-Src, which stimulates the proliferation of endocrine-resistant breast cancer cells. In the nucleus, E2 activates classical transcription pathway ERE, which is involved in cellular proliferation. Simultaneously, E2 consistently activates the tethering pathway of ERα, particularly AP-1 family members. This leads to stress responses in the endoplasmic reticulum. Figure republished with permission from AACR (49).

The oncogene c-Src is closely associated with the ER, which is activated by E2 in LTED breast cancer cells (47). Inhibition of c-Src tyrosine kinase blocks E2-induced apoptosis (47, 48). Similar to endocrine-sensitive breast cancer cells, c-Src plays a critical role in the mediation of non-genomic pathways of the ER activated by E2, as well as by EDC in the LTED breast cancer cells (47) (**Figure 1**). The proliferative action of EDC allowed the exclusion of the nongenomic action of c-Src in E2-induced apoptosis (47). Further findings demonstrated that c-Src is involved in the activation of stress pathways by E2, which results in apoptosis (47, 50) (**Figure 1**). The inhibition of cSrc prevents the activation of stress pathways, which subsequently block estrogen-induced apoptosis. A cluster of stress-related genes, which are regulated by both the ER and c-Src and determine the fate of these LTED breast cancer cells, were screened out by RNA-sequencing (47).

During the progression of E2-induced apoptosis in LTED breast cancer cells, there is accumulation of various stress responses, including endoplasmic reticulum stress, oxidative stress and inflammatory stress (47, 51–53). These stress responses are highly interrelated processes (47, 51), however, they occur at different time points during E2 treatment. The unfolded protein response (UPR) is initially activated by E2 in the endoplasmic reticulum after only hours of treatment, which include the activation of three sensors- PRK-like endoplasmic reticulum kinase (PERK), inositol-requiring 1 alpha (IRE1α), and activating transcription factor 6 (ATF6), all of which have different functions (47, 54). The PERK phosphorylates eIF2α to attenuate protein translation, while IRE1α and ATF6 mainly mediate endoplasmic reticulum stress-associated degradation (ERAD) of PI3K/Akt/mTOR pathways (54). The release of reactive oxygen species (ROS) and oxidative stress indicator HMOX1 are activated after 72 hours of E2 treatment (47). As for the apoptosis-associated inflammatory factors, TNF family members are expressed in a delayed pattern that peak after 72 hours of E2 treatment (47). Undoubtedly, PERK is a critical molecule to regulate oxidative stress and TNFα expression, thereby determining cell fate (47, 49, 54).

**Figure 2:**
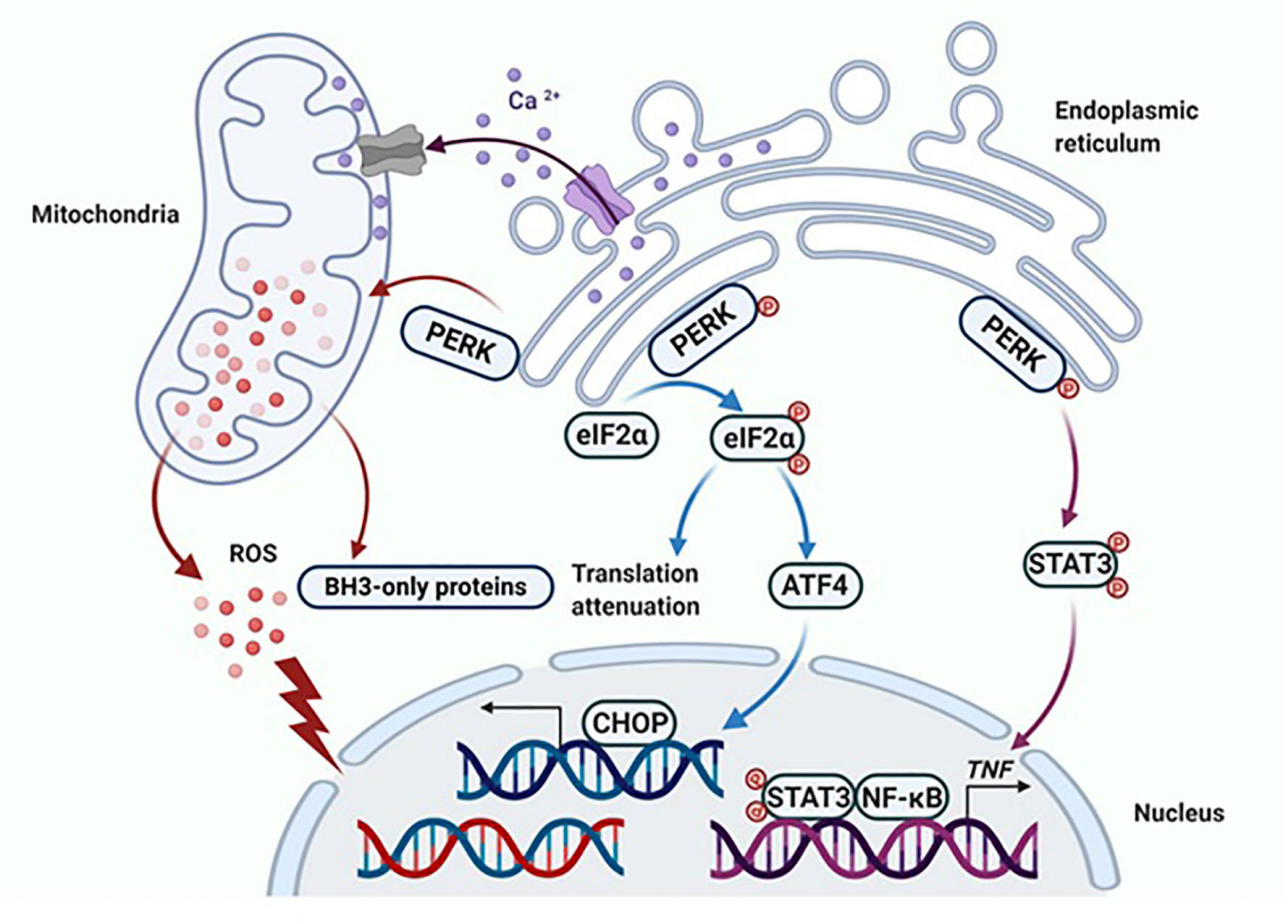
PERK activates apoptotic cascades in multiple ways. Apoptosis is ultimately induced by sustained PERK activation after E2 therapy in endocrine-resistant breast cancer cells. The classical way that PERK induces apoptosis is by activating ATF4 and the downstream pro-apoptotic protein CHOP. Additionally, PERK regulates the function of the mitochondria and results in the release of ROS and overexpression of BH3-only proteins. Finally, the extrinsic apoptotic pathway is activated by PERK that is mediated by STAT3 to increase the DNA-binding activity of NF-κB and subsequently induce TNFα expression. Figure republished with permission from AACR (49).

Specifically, sustained activation of PERK phosphorylates eIF2α, which induces activation of ATF4 and the downstream pro-apoptotic protein CHOP (55). Nevertheless, this apoptotic effect of PERK is not solely dependent on the phosphorylation of eIF2α (49). Continuous activation of PERK leads to mitochondrial fragmentation, increased reactive oxygen species (ROS) production and overexpressed BH3-only proteins (**Figure 2**) (47, 49). Furthermore, PERK participates in the regulation of inflammatory responses, in particular, involving in the E2-induced TNFα expression, which relies on PERK for the activation of NF-κB (**Figure 2**) (49, 56, 57). The PERK phosphorylates the stress-associated transcription factor STAT3 and promotes its translocation to the nucleus. The STAT3 facilitates activation of NF-κB and the induction of TNFα expression, ultimately leading to apoptosis (56, 57). It was determined that the PERK/NF-κB/TNFα axis plays a critical role in inducing apoptosis after E2 treatment (56, 57), and hence, PERK plays multiple roles in the activation of apoptotic cascades (49).

The key role of NF-κB in E2-induced apoptosis creates new opportunities to modulate its DNA-binding activity through other nuclear transcription factors, such as the glucocorticoid receptor (GR) (58) and the peroxisome proliferator-activated receptor γ (PPARγ) (59), thereby, affecting E2-induced apoptosis (56–59). These particular findings are used to interpret the results of the aforementioned WHI study demonstrating a decrease in breast cancer incidence in women taking conjugated equine estrogen (CEE) alone as hormone replacement therapy (HRT) (29, 30). A synthetic progesterone medroxyprogesterone acetate (MPA) has glucocorticoid activity (60) that reverses the anticancer effect of E2 and increases breast cancer incidence in postmenopausal women (30, 60, 61). In particular, MPA activates the GR, which suppresses the DNA-binding activity of NF-κB, thereby, blocking E2-induced apoptosis in the LTED breast cancer cells (58, 61). This topic has been covered in detail in a recent review (61).

To summarize, the nuclear ER is the initial site for E2 induction of apoptosis in the LTED breast cancer cells (47, 49). Activation of the nuclear ER leads to stress responses in these cells (47, 49, 51). Subsequently, multiple stress-associated transcription factors are involved in this process and two major organelles- endoplasmic reticulum and the mitochondrion- are undergoing stress responses after E2 treatment in these endocrine-resistant breast cancer cells (30, 47, 49, 54, 56–61). The PERK conveys signal between nucleus and cytoplasm to determine cell fate (49, 56, 57). All of these findings provide an important rationale for investigators to design novel strategies on how to appropriately modulate the UPR to improve the therapeutic effects of E2-induced apoptosis on endocrine-resistant breast cancer. However, the logical question would be how is estrogen-induced apoptosis dependent on the structure of the estrogenic ligand that binds to the ER?

## Structure-Activity Relationship of the ER and Estrogen-Induced Apoptosis

**Figure 3:**
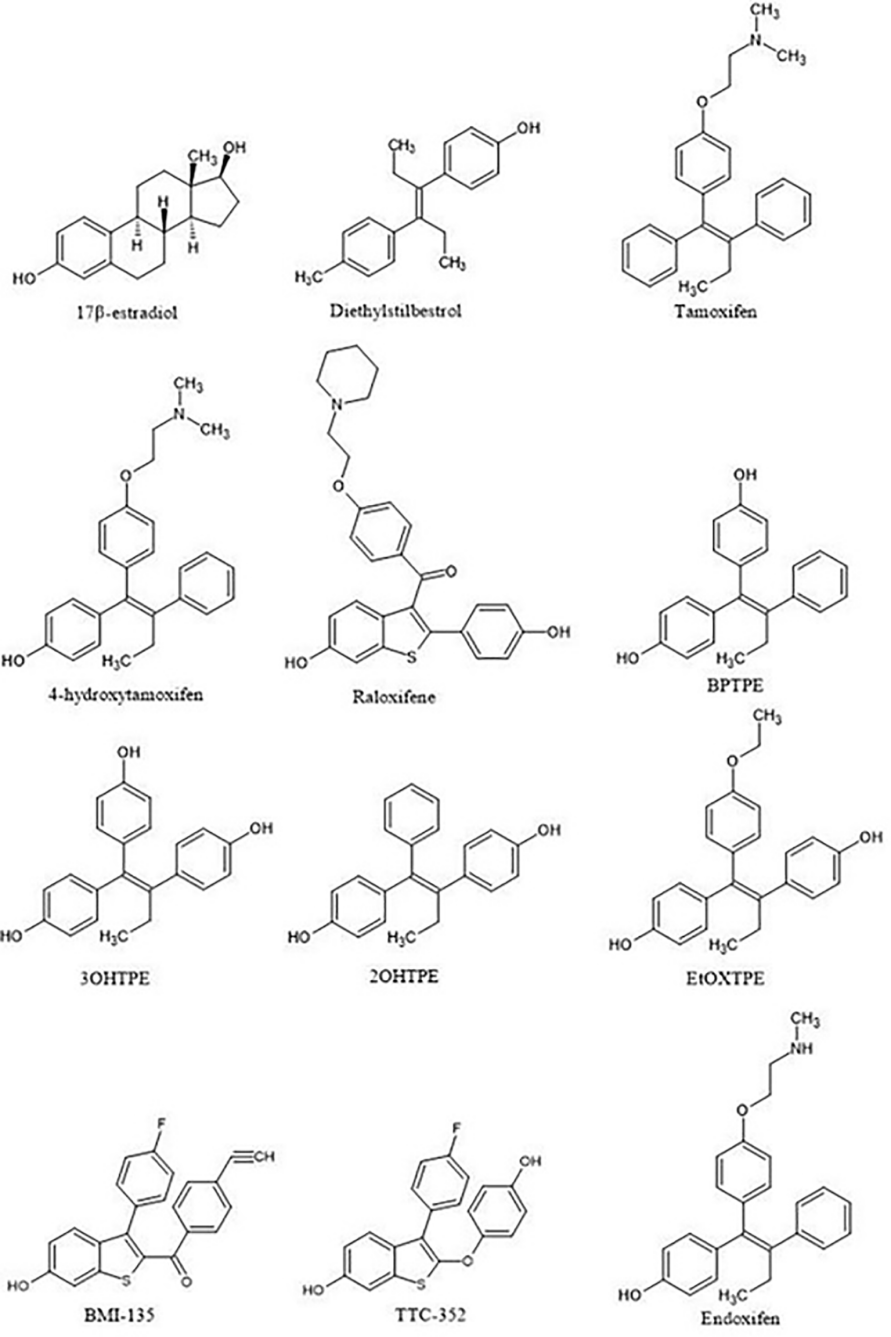
Compounds used in the structure-function relationship studies over the 15 years to assess the role of ligand:ER in the modulation of the estrogen-induced apoptosis.

It is appropriate to start with the first studies of the structure-function relationships of the ER ligand with the activity of the receptor. The first SAR studies were conducted to decipher the mechanism of action of non-steroidal antiestrogens (tamoxifen (**Figure 3**) and its derivatives) with the ER (62–67). These studies are important as they were the first SAR investigations that hypothesized the existence of a region in the ER called then the Anti-Estrogenic Region (62), which was thought to be responsible for the interaction with the alkylaminoethoxy side chain of tamoxifen to produce an antiestrogenic conformation of the receptor and block estrogen action, in accordance to the Belleau’s conformational theory. A decade later, in the course of the *in vitro* investigation of the mechanisms of acquired resistance to tamoxifen during long-term treatment, a point substitution mutation in the ER was discovered at codon 351 (68). The mutation in amino acid aspartate to tyrosine at position 351 (Aps351Tyr) was present in a tamoxifen-stimulated MCF-7 variant after long-term tamoxifen exposure and was located in helix 12, a part of the Ligand-Binding Domain (LBD).

**Figure 4:**
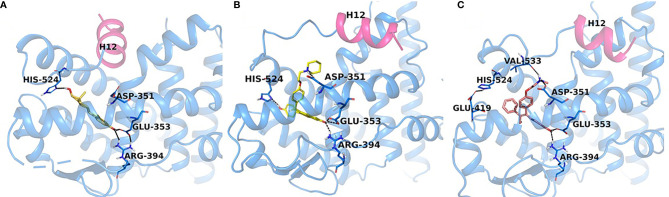
Experimental structures of ERα-LBD in complex with E2 (PDB code: 6CBZ), agonist conformation **(A)**, raloxifene (PDB code: 1ERR), **(B)** and endoxifen (PDB code: note assigned yet), **(C)** antagonist conformations. H12 (magenta) seals E2 (orange) in the binding pocket, while in the antagonist conformation H12 is shifted to accommodate the Iigand, RAL (yellow) and Endoxifen (pink) which interact with Asp351.

It should be noted that the ER itself was not yet crystallized, but the LBD of the ER would be crystallized with E2 and raloxifene (**Figures 3**, **4**) (69) in 1997, although the first domain of the ER to be crystallized was the DNA binding domain (70) in 1993. This crystallization of the LBD is important because it helped to position Asp351 in a three-dimensional model. As mentioned previously, helix 12 acts as a “lid” over the ligand-binding cavity, allowing an agonist such as E2 to be enclosed in the ligand-binding cavity and for the receptor to take an agonist conformation to be activated (**Figure 4**). At the same time, antiestrogenic side chain of raloxifene that was used as an antiestrogenic ligand for crystallization (69) interacts with Asp351 and shields it inducing an antagonist conformation of the receptor, which prevents its activation (**Figure 4**). This was determined in a study earlier investigating the role of the naturally occurring mutation Asp351Tyr in ER-negative MDA-MB-231 breast cancer cell line stably transfected with DNA constructs for expression of either wild-type ER or the Asp351Tyr mutant ER and the endogenous TGFα reporter gene (71). The mutation of Asp351 residue reversed the antiestrogenic properties of raloxifene, meanwhile E2’s ability to activate the receptor was not affected by the mutation, neither was fulvestrant’s (a pure antiestrogen) ability to degrade the receptor. A follow up study (72) expanded the evidence to support the model that the antiestrogenic side-chain was a “stick in the jaws of crocodile” (62, 71–73). Subsequently, it was demonstrated that Asp351Gly substitution mutation completely abrogated low estrogenic properties of tamoxifen (full estrogenic properties in case of the endogenous TGFα reporter gene) in a similarly transfected ER-negative MDA-MB-231 breast cancer cells with ER-expressing constructs (74). This phenomenon was explained by the fact that the raloxifene’s piperidine ring was neutralizing the charge on Asp351 and alkyaminoethoxy side chain of tamoxifen could not and, thus, exhibited a different pharmacological property. A very important part of the mechanism of the ER activation is the binding of the Steroid Receptor Coactivators (SRCs) to the external surface of the receptor protein. This same study demonstrated that triple mutation in the AF2 region of the receptor (Asp538Ala, Glu542Aala, and Asp545Ala) reduced the activation of the receptor by E2 and tamoxifen, indicating that both AF1 and AF2 regions are necessary for the ER activity.

Ultimately, this led to the investigation of the role of Asp351 amino acid residue in the activation of the ER by two different types of estrogens: planar and angular (75). It was demonstrated in previously described ER-negative breast cancer cells stably transfected with DNA constructs expressing wild-type or mutant ER that planar estrogens, such as E2 and DES (**Figure 3**) (which has been used on Sir Alexander Haddow’s clinical trials in the 1940’s), activated the ER regardless of the Asp351 mutational status. However, this is opposite for the angular estrogenic TPE derivative (fixed-ring 4-hydroxytamoxifen without a side chain), which was used as an angular estrogen. The angular TPE required the interaction with Asp351 and the mutation prevented the activation of the ER and thus the activation of the TGFα reporter gene. This region containing the 351 amino acid residue was called the AF2b. A molecular classification of estrogens based on their three-dimensional structure and their ability to interact with Asp351 for the ER activation was proposed (75). Class I estrogens are planar estrogens and do not require Asp351 to activate the ER and Class II estrogens and angular and require interaction with Asp351 for the receptor activation. By the time estrogen-induced apoptosis was established as a reproducible clinically relevant phenomenon, a question arose whether estrogen-induced apoptosis is dependent on the class of estrogen activating the ER.

**Figure 5:**
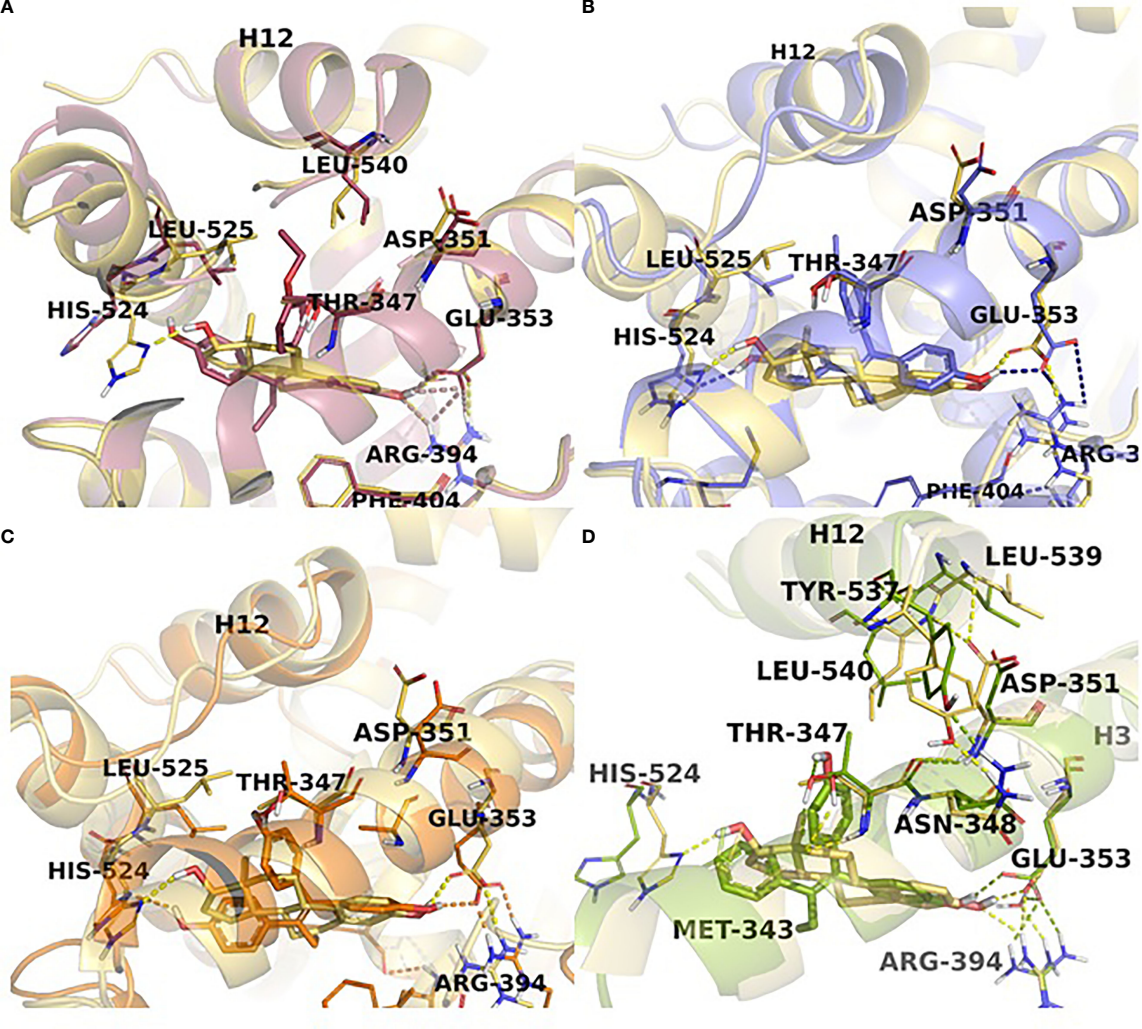
TPE derivatives in the receptor active site. Close view representations of ERα-E2 (yellow) superimposed with **(A)** EtOXTPE (pink), **(B)** Z2OHTPE (purple), **(C)** 3OHTPE (orange), **(D)** BPTPE (green). Snapshots extracted from the MD trajectories are depicted for each receptor-ligand complex. The key interactions in the active site are shown (in dash lines) together with the positioning of helix12 in comparison with the ER:E2 structure.

The first experiments in the MCF-7:5C LTED breast cancer cells that undergo estrogen-induced apoptosis demonstrated that within the same time frame in which E2 induces apoptosis (7 days) does not work for triphenylethylene derivatives with a free hydroxyl group in the para-position (76). It has been previously determined (77) that the same TPE derivatives (**Figure 3**) are fully estrogenic in the wild-type MCF-7 cells and are able to activate the ER in these cells and promote growth. Indeed, the TPE derivatives required Asp351 to activate the ER, Asp351Gly mutation prevented the activation of the ER by the TPEs. At the same time, E2 was able to activate both wild-type and mutant types of the ER. However, as previously stated, TPEs with a para-hydroxyl group did not induce efficient estrogen-apoptosis within 7days, whereas planar E2 did. In fact, some of the TPEs, bisphenol TPE (BPTPE), 3OHTPE (a trihydroxylated derivative of TPE) (76, 78) induced partial/minor estrogen-induced apoptosis within 7 days of treatment and EtOXTPE (a 4-hydroxylated TPE with an ethoxy side chain in the para-position) did not. Interestingly, only Z2OHTPE (a trans-isomer of dihydroxylated TPE) was able to produce a full apoptotic effect as well as E2 in these cells (79). Additionally, the TPEs with a para-hydroxyl group were able to inhibit E2-induced apoptosis within the first week of treatment, similar to 4OHT in MCF-7:5C cells consistent with their intrinsic pharmacologic activities. Normally, E2 would induce 26S-proteosome degradation of the ER in order to sustain the transcriptional activity of the target genes, these TPEs practically did not, which is more similar to 4OHT (76). Chromatin Immunoprecipitation assays (ChIP) showed that the TPEs did not recruit SRC3 steroid nuclear receptor coactivator very well to the ER complex on the estrogen-dependent gene promoter (76, 78–80) and only partially recruited the ER proteins to the promoter regions of estrogen-dependent genes, such as TFF1 and GREB1. However, Z2OHTPE was very close to E2 in the levels of recruitment. Molecular docking modelling revealed that the TPEs can create a hindrance for helix 12 in proximity of the Asp351 residue and create a conformation of the receptor more similar to 4OHT : ER complex than to E2:ER complex (**Figure 5**) (76–78). The initial conclusion was that the conformation of the ER was less demanding for inducing growth of wild-type breast cancer cells, but much more so for the induction of apoptosis *via* activation of the receptor in LTED breast cancer cells (76). When comparing the pharmacology of BPTPE and a planar environmental estrogen bisphenol-A (BPA) it further underscored the major role of Asp351 in the activation of the ER by planar and angular estrogens (78). Activation of the ER by planar BPA did not depend on the mutational status of Asp351, whereas for angular BPTPE, Asp351 was crucial, identical to the previous results (76). Both types of estrogens were able to induce growth in wild-type MCF-7 breast cancer cells, but only planar BPA was able to induce apoptosis effectively in MCF-7:5C LTED breast cancer cells within 7 days of treatment, similar to E2, when compared to BPTPE. It was also shown that the induction of pro-apoptotic genes in the LTED breast cancer cells by BPTPE was more similar to 4OHT profile, unlike BPA, which was more similar to E2. This was consistent with ChIP assay results, which showed that BPA was a full agonist in ability to recruit the ER and SRC3 co-activator to the estrogen-dependent TFF1 gene promoter (78). Conformation of the ER obviously mattered when it came to estrogen-induced apoptosis. This was consistent across practically the whole panel of angular TPE derivatives (81). However, it was quickly discovered that TPEs (BPTPE and EtOXTPE, in particular) are able to induce ER-mediated apoptosis after more than a week of treatment (80, 82). It was demonstrated that BPTPE has a different rate of “commitment” of the cells to estrogen-induced apoptosis when compared to E2, which is in itself a delayed process when compared to cytotoxic agent-induced apoptosis like paclitaxel (83). When compared to E2, LTED cells treated with BPTPE took longer to “commit” to apoptosis before the cells could be “rescued” with 4OHT (82). In fact, if MCF-7:5C cells were treated with BPTPE for 5 days or more then the gene expression profile changed when compared to control or 4OHT treatment for both cell cycle regulating genes (no checkpoint blockade with increased S-phase similar to E2) and apoptotic genes. As noted in the mechanisms of estrogen-induced apoptosis section, Endoplasmic Reticulum Stress (ERS) plays a crucial role. Inhibition of caspase-4, a caspase induced during ERS (84), which was also found to be upregulated in E2-treated MCF-7:5C cells in microarray studies (51), was able to block BPTPE-induced apoptosis. The BPTPE, in fact, does induce upregulation of inflammatory stress (IS) genes and ERS-related genes, however, unlike E2, which does it after 48 hours, BPTPE induced these genes only after 4 days of treatment. This is consistent with the result of delayed apoptosis by an angular estrogen BPTPE.

A 4-hydroxylated TPE derivative with an ethoxy side chain in para-position called EtOXTPE was also tested (80) in the same LTED MCF-7:5C breast cancer cell line and demonstrated a consistent delayed apoptosis compared to E2. In wild type MCF-7 cells the EtOXTPE is a full agonist, promoting cell growth, with low binding affinity for the ER (77), however, only a partial agonist in MCF-7:5C cells. The compound was not able to induce efficient apoptosis in the first week of treatment at all but induced full ER-mediated apoptotic effect within 2 weeks of treatment. At the same time, in the first week of treatment EtOXTPE was able to inhibit E2-induced apoptosis, similar to other TPE derivatives with a para-hydroxyl group. Similar to these TPEs, it took EtOXTPE longer to induce pro-apoptotic gene upregulation in the cells compared to E2. It was previously demonstrated that EtOXTPE did not downregulate the ER protein in these cells like E2 did, but was more similar to 4OHT in the first 24 hours and took longer to downregulate the ER protein (76, 80). However, it was discovered that EtOXTPE was able to downregulate ESR1 mRNA levels over time similar to E2 (80). As mentioned above, UPR plays an important role in estrogen-apoptosis. It was previously determined that UPR genes are upregulated during E2 treatment of MCF-7:5C cells (51), and that inhibition of PERK, which mediates a part of the UPR in the cell, abrogates estrogen-induced apoptosis (47). As mentioned above, PERK is upstream of eIF2α and facilitates the phosphorylation of the latter during UPR stress. It was found that EtOXTPE is also able to induce the phosphorylation of eIF2α similar to E2, although a bit later. However, in combination with antiestrogen endoxifen [a biologically active metabolite of 4OHT (**Figure 3**)] EtOXTPE is less potent in the induction of phosphorylation of eIF2α compared to E2, which is consistent with the intrinsic biological activity of the compound. Nevertheless, inhibition of PERK in MCF-7:5C cells was able to inhibit EtOXTPE-induced apoptosis at later time points. The compound also was a partial agonist in regards to recruitment of the ER : SRC3 complex to the promotor region of estrogen-dependent gene TFF1 (76) and also depended on Asp351 for activation of the receptor (77). Although molecular docking was performed previously using the EtOXTPE structure, X-ray crystallography was performed to validate this hypothesis (**Figure 5**) (80).

The resolution of the three-dimensional structure of the EtOXTPE : ER LBD complex obtained from X-ray crystallography (**Figure 5**) revealed that the compound induced a novel conformation of the receptor’s LBD. It should be noted that EtOXTPE isomerizes in solution and both trans- and cis-isomers were present in the crystallography procedure and all biological experiments, however, it was found that only trans-isomer was present in the crystallized LBD of the ER. The TPE derivative formed bonds with Glu353 and Arg394 with its first phenol ring, similar to the analogous ring of E2. However, that is where the similarities end. Amino acid residue His524 was forced to the external surface of the LBD when bound with EtOXTPE, which ultimately prevents the formation of the hydrogen bond with EtOXTPE, unlike with E2. This particular amino acid is a part of the H-bond network that a ligand like E2 forms within the ligand-binding cavity of the receptor, that captures E2 inside and provides stability to the agonist conformation of the ER:E2 complex. Since this H-bond is not formed between His524 and EtOXTPE, this affects the stability of the agonist conformation of the receptor. Instead, His524 forms hydrogen bonds with Gly521 and Lys520, while the amino group of Lys520 forms a bridge salt with the carboxylate group of Glu523. The side chains of Leu525 and Leu540 are shifted to accommodate and participate in Van Der Waals interactions with the ethoxy side chain of EtOXTPE, however, Asp351 is close by. Together, these changes perturbate the orientation of helix 11 such that it is now closer to helix 12. This demonstrated that ER can adapt an agonist conformation in alternate fashion to E2.

Despite the presence of an ethoxy side chain on EtOXTPE derivative, the compound was able to induce the activation of the ER and ultimately estrogen-induced apoptosis in MCF-7:5C LTED breast cancer cells. However, as mentioned before, a compound called Z2OHTPE, which has no groups in the para-position, was able to produce an E2-like effect in the same time frame (79). The compound did not act as an antiestrogen in the first week of treatment, preventing E2-induced apoptosis like a TPE with a free hydroxyl group in the para position, such as BPTPE, as it is a full agonist. Microarray studies showed that Z2OHTPE was more like E2 in the global gene expression profile regulation than BPTPE and antiestrogen endoxifen and was able to induce pro-apoptotic genes much quicker in MCF-7:5C cells than BPTPE or 3OHTPE. Despite the fact that Z2OHTPE is able to activate the transcription of estrogen regulated genes like TFF1 and GREB1 as well as E2 and recruit ER protein to their promoter regions similarly to E2, as demonstrated by ChIP assays, the compound still was lagging in the recruitment of SRC3. Subsequent ChIP assays with masspectrometry identification of co-activators and co-repressors revealed that Z2OHTPE was almost identical to E2 in the recruitment of various coactivators (for example p160/steroid receptor coactivator (SRC) family members (NCOA3, but not NCOA1 and 2), NCOA6, p300 (EP300), and the Mediator complex (MED subunits), in addition to KMT2C/2D histone methyltransferases). compared to BPTPE and 3OHTPE, which recruited coregulators more similar to antiestrogen endoxifen (for example RBM39 and MYBL2 with BPTPE; PHC3 and TRIM28 with 3OHTPE). Essentially, the only structural difference between Z2OHTPE and other TPE derivatives is the absence of a free hydroxyl on the para-phenol ring. This dictates a variation in the agonistic conformation of the ER bound with Z2OHTPE and other TPEs and thus the ability to recruit certain activating coregulators. For this reason, X-ray crystallography of the ER LBD bound with E2, endoxifen, BPTPE, 3OHTPE and Z2OHTPE was performed.

The conformations of the ER LBD with either TPE derivative was a canonical agonist conformation with H12 sealing the ligand-binding cavity, similar to E2, however, with some minor differences (**Figure 5**). The first phenol ring of all the TPEs formed the H-bond with Glu353 and Arg394 and is identical to the analogous ring of E2. The additional phenolic hydroxyl of Z2OHTPE and 3OHTPE forms H-bonds with His524, like E2, whereas a feature specific to 3OHTPE and BPTPE is the formation of an H-bond with Thr347, which in turn connects to Asp351 that is essential for the ER activation by class II estrogens. The hydrophobic interactions account for the remaining contacts with the binding pocket. Because of the technological limitations, minor differences in the crystal structures for Z2OHTPE : ER complex and complexes with other TPE corresponding to their respective biologic properties could not be resolved. Thus molecular dynamics simulations were performed.

Molecular dynamic simulations showed that there are certain differences in the interaction of the ligands and the ER that modulate the stability of the complexes and, thus, could affect the transcriptional activity of the receptor (**Figure 5**). It was determined that the least stable TPE : ER complex is with BPTPE, which creates an increased flexibility of the various helices in the LBD. Interestingly, Z2OHTPE creates a considerably more frequent interaction with His524 in the ligand-binding cavity, which is similar to E2. Other TPE derivatives formed this H-bond considerably less frequently, which can explain partial agonist activity of these compounds in regards to the induction of apoptosis in MCF-7:5C LTED cells. This interaction with His524 induces stability of the complex with the ER through the hydrophobic contacts with Leu525. There are some interactions with amino acid residues that are unique to TPEs, for example interaction with Thr347 and the phenolic group of 3OHTPE and BPTPE occur 90% of the time. All the simulation results indicate that the TPEs form unique conformations *via* unique H-bonds and hydrophobic interactions with amino acid residues that can alter the stability and, thus, positioning of helices 3 and 11 that constitute the receptor’s LBD and consequently displaces helix 12 and the coactivator binding site. The most notable displacement was with BPTPE, followed by 3OHTPE and the least with Z2OHTPE, which is much more similar to E2 than the others. It can be presumed that the less stable binding of BPTPE to the ER is due to the reduced interaction with His524. Another residue that Z2OHTPE and E2 frequently interact with is Tyr537, which forms an H-bond with Asn348, a residue right next to Thr347 (**Figure 5B**), which, as mentioned above, uniquely forms an H-bond with 3OHTPE and BPTPE only. In the case of the later, Thr347 is drawn closer to the ligands and this creates additional instability of the complexes and displacement of H12, whereas with Z2OHTPE it does not interact with this residue and the interactions with Asn348 and Tyr537 are not affected with E2. Altogether, these results demonstrate that the structure of an angular estrogen, in particular the presence or absence of a free hydroxyl in para-position can affect the conformational stability of the TPE : ER complexes that, in turn, affects the ability of H12 to “close” and bind the co-activators and produce a full agonist effect, such as estrogen-induced apoptosis in LTED breast cancer cells.

**Figure 6:**
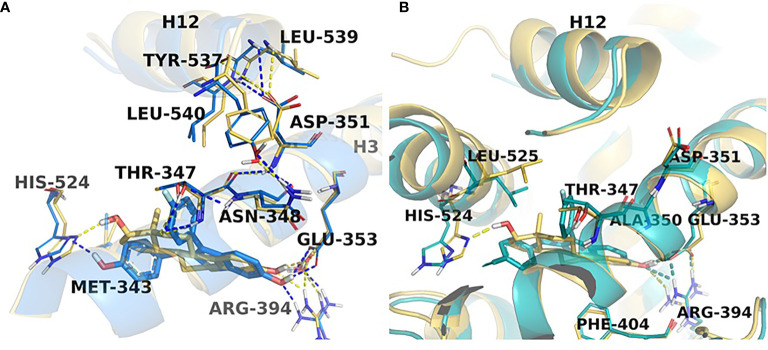
ShERPA derivatives in the receptor active site. Close view representations of ERα-E2 (yellow) superimposed with **(A)** TTC-352 (blue) and **(B)** BMI-135 (cyan). Snapshots extracted from the MD trajectories are depicted for each receptor-ligand complex. The key interactions in the active site are shown (in dash lines) together with the positioning of helix12 in comparison with the ERα-E2 structure.

The peculiarities of the angular estrogen interactions with the ER LBD and its effect on estrogen-induced apoptosis were further evaluated with the use of novel non-steroidal estrogens called Selective Human Estrogen Receptor Partial Agonists (ShERPAs) (85). Two ShERPAs called BMI-135 and TTC-352 (**Figure 3**) were tested alongside with E2 and BPTPE (86, 87). Compound TTC-352 has completed successfully phase I clinical trial for treatment of heavily pretreated hormone refractory breast cancer (88). Both ShERPAs have a naphthalene core but with a less bulky antiestrogenic side chain. Evaluation of the biological activity of both ShERPAs demonstrated that both are actually full agonists, inducing growth of wild-type MCF-7 cells and estrogen-induced apoptosis in MCF-7:5C, MCF-7:2A and MCF-7:RAL LTED breast cancer cells, although, with less potency as they required higher concentration to achieve full agonist activity. Estrogen-induced apoptosis in MCF-7:5C cells was observed after one week of treatment with both ShERPAs similar to E2 and after 2 week of treatment in MCF-:7:2A cells and MCF-7:RAL cells, which is consistent with the induction of apoptosis in all these cell lines with E2. At the same time, both ShERPAs activated transcriptional activity of estrogen-responsive genes such as TFF1 and GREB1 in LTED breast cancer cell lines and wild type cells at the same levels as E2. Moreover, both ShERPAs recruited the same levels of ER protein to the promoter region of TFF1 and GREB1 genes as E2, however, they recruited on average less SRC3 than E2 (86, 87). Importantly, both ShERPAs activated the UPR in MCF-7:5C LTED cells before the induction of apoptosis. This was proven by detection of the downregulation of pro-survival UPR-associated genes *via* UPR RT-PCR gene profilers (86) and the activation and upregulation of pro-apoptotic UPR-associated protein markers such as phosphorylated eIF2α, CHOP, ATF4 (87) as well as live-cell staining of MCF-7:5C cells with thioflavin T, a specific dye for ERS (86, 87). Inhibition of PERK pathway, mentioned above, was able to abrogate the estrogen-induced apoptosis for both ShERPAs and inhibition of IRE1α:XBP1 pathway enhanced the apoptosis (87). Antiestrogen 4OHT was able to block apoptotic action of both ShERPAs indicating that the action is mediated by the ER. Additionally, both ShERPAs recruited same coregulators as E2, while BPTPE recruited coregulators more similar to endoxifen (87) in both wild-type and MCF-7:5C LTED cell lines, which is consistent with previous results obtained from cell-free DNA pulldowns with liquid masspectrometry (79). However, there were some differences indicative of perturbations in agonist conformation of the ER complex liganded with the ShERPAs. These differences were the inability of the ShERPAs to recruit SRC1 and SRC2 compared to E2 and higher recruitment of MED coregulator subunits than E2 in MCF-7:5C cells. To compare and contrast the conformations of the ShERPA : ER complexes with E2 complexes X-ray crystallography and molecular docking with molecular dynamics simulations were performed (**Figure 6**).

X-ray crystallography for TTC-352 bound to the ER LBD was performed and the resolved structure has revealed an agonist conformation of the receptor’s LBD with helix 12 “closed” over the ligand-binding cavity (**Figure 6A**) (87). Very minor deviations were found in the agonist conformation of the receptor compared to E2 complex experimental structure. The TTC-352 shared the same H-bond network with Phe404, Glu353 and Ard394 as E2. When compared to BPTPE, as previously noted, BPTPE does not form an H-bond with His524, which is common for both E2 and TTC-352. Also, BPTPE displaces Tyr537 *via* the interaction with Thr347 with its second phenyl ring, which TTC-352 or E2 do not. This repositioning at the base of helix 12 reorients Leu540 and does not allow for the interaction with Asp351, Leu539 and Leu540 for the receptor’s conformation stabilization, which is not the case for TTC-352. Molecular dynamic simulations revealed that, in fact, TTC-352 produces a more stable agonist conformation of the ER LBD and positioning of helix 12 with low volatility when compared to BPTPE, which can explain the compound’s full agonist activity in the LTED breast cancer cells (87). It was determined that the interaction of TTC-352 with Glu353 is the driving force behind the stability of the receptor’s conformation in the molecular dynamic simulations, whereas this interaction is much weaker for BPTPE.

Using the same experimental structure of the ER’s LBD with TTC-352, BMI-135 was docked (86) (**Figure 6B**). The compounds shared the same network of H-bonds as E2, in particular, Glu353 and Arg394 and other amino acid residues shared with E2’s A and B rings. Interestingly, unlike E2 and TTC-352, BMI-135 did not form an H-bond with His524, which is more similar to BPTPE. Molecular dynamic simulations showed that despite the fact that BMI-135 did not form an H-bond with His524, occasional interactions were still observed *in situ*. A striking difference was noticed for BMI-135, which displayed the largest peak of side-chain root-mean-square fluctuation (RMSF, a measure of volatility of the structure) for Arg394. This mobility indicated that Arg394 was not involved in a direct H-bond with the ligand and/or ionic bridges to Glu353, therefore not stabilizing it. These differences indicated yet another alternative for the ER LDB to adopt an agonist conformation dependent on the structure of the ligand and ultimately trigger estrogen-induced apoptosis in LTED breast cancer cells.

## Conclusion

Over the past decade our group has deciphered the molecular mechanisms of estrogen-induced apoptosis and established the role of the structure of the ER ligand in the induction of this phenomenon. It has become apparent that not only planar steroid estrogens can induce apoptosis in LTED breast cancer cells but also certain non-planar estrogens. This SAR is dependent on positioning of substituted groups on the ligand and its interaction with the ligand-binding pocket of the ER, which, in turn, can perturbate the positioning of helix 12 in a stable agonist conformation and activate the receptor. This has clinical relevance as the use of non-steroidal estrogens may be more desired in terms of side effects as has been described in earlier clinical trials in the 1950’s (89). Induction of estrogen-induced apoptosis in LTED breast cancer may become a feasible and safe alternative therapy for many patients with lower side effects for many patients in the future.

## Author Contributions

PM wrote and edited the manuscript prepared **Figure 3**. PF wrote a part of the manuscript and made **Figures 1** and **2**. RC made **Figures 4**–**6**. BA contributed data for the review. VCJ edited the review. All authors contributed to the article and approved the submitted version.

## Funding

VCJ thanks the George and Barbara Bush Endowment for Innovative Cancer Research and the benefactors of the Dallas/Fort Worth Living Legend Chair for Cancer Research for their generous support. This work was supported by the NIH/NCI under award number P30-CA016672 (P.W. Pisters), Susan G. Komen for the Cure Foundation under award number SAC100009 (PJ), and Cancer Prevention Research Institute of Texas (CPRIT) for the STARs and STARs plus Awards (VCJ). Coriolan Dragulescu Institute of Chemistry of the Romanian Academy (Project no. 1.1/2022) (RC).

## Conflict of Interest

The authors declare that the research was conducted in the absence of any commercial or financial relationships that could be construed as a potential conflict of interest.

## Publisher’s Note

All claims expressed in this article are solely those of the authors and do not necessarily represent those of their affiliated organizations, or those of the publisher, the editors and the reviewers. Any product that may be evaluated in this article, or claim that may be made by its manufacturer, is not guaranteed or endorsed by the publisher.
